# Deep-Learning-Assisted Single-Shot Plasma Emission Imaging for Pollen Morphology Reconstruction and Apparent Hydration-State Classification

**DOI:** 10.1021/acsomega.6c07927

**Published:** 2026-09-12

**Authors:** James A. Grant-Jacob, Yuchen Liu, Ben Mills

**Affiliations:** 7423 University of Southampton, Optoelectronics Research Centre, Southampton SO17 1BJ, GB

## Abstract

Monitoring pollen is important for crop productivity, plant breeding, and environmental forecasting, yet current approaches remain limited in throughput and physiological insight. Here, we combine single-shot plasma emission imaging with artificial intelligence for pollen morphology reconstruction and apparent hydration state classification. Plasma plumes generated during laser–pollen interaction provide indirect measurements of pollen morphology, extending plasma-based analysis beyond conventional spectroscopic readouts. A conditional generative adversarial neural network reconstructed morphology from such plasma images, while chromaticity analysis revealed systematic color shifts between hydrated and dehydrated grains. A support vector machine using chromaticity histogram and scalar color features distinguished these groups with ∼76% leave-one-out accuracy. The results suggest that plasma emission contains structural and hydration-associated information, supporting rapid pollen morphology inference and physiological phenotyping for agricultural and environmental monitoring.

## Graphical abstract

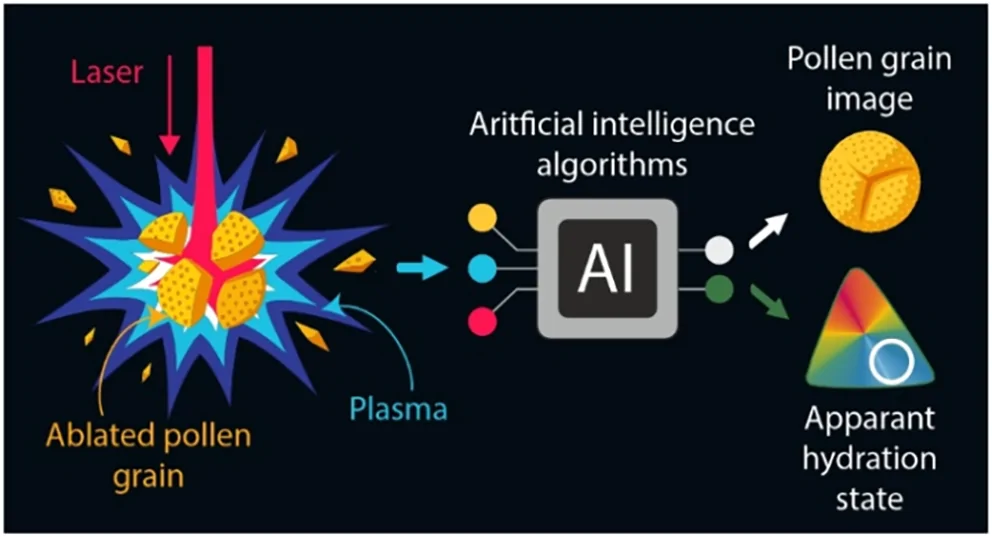

## Introduction

Pollen viability is a critical determinant of fertilization success, crop yield, and ecosystem function,^1–6^ yet rapid and scalable methods for characterizing pollen morphology and physiological state remain limited.^7–9^ In addition to their agricultural and environmental importance, pollen-derived structures are increasingly being investigated as functional biomaterials and components of bioinspired microfluidic systems.^10–12^ These developments further motivate methods capable of rapidly characterizing the morphology and condition of pollen and other biological microparticles. Currently, conventional assessment relies on microscopy or methods that are low-throughput and incompatible with real-time or large-scale field deployment.^13,14^ These conventional pollen monitoring techniques generally require manual sample preparation, microscopy, counting, and interpretation by trained users, commonly producing 24 h aggregated measurements rather than real-time information. While automated optical systems can provide hourly or real-time measurements of pollen grains, such imaging setups are often comparatively complex and require specific classification pipelines.^15^ New sensing modalities capable of extracting information on pollen structure and physiological state from indirect optical signatures, rather than from conventional high-resolution morphological inspection alone, would represent a significant advance for precision agriculture and plant breeding.

Laser-induced breakdown spectroscopy (LIBS)^16–19^ and related plasma emission techniques^20^ have demonstrated sensitivity to the elemental and molecular composition of biological materials. However, previous pollen-focused studies have primarily emphasized spectroscopic identification or compositional analysis,^21–23^ thus using only part of the information available in the laser-induced breakdown plasma signal.

In particular, plasma emission also contains spatial and morphological information arising from the interaction between the focused ultrashort laser pulse and the particle. Recovering this underexploited information requires analytical approaches capable of learning complex mappings from transient optical signals to biologically meaningful outputs.

Deep learning,^24–27^ and specifically image-to-image translation neural networks,^28^ have become prevalent in research in recent years owing to their ability to learn complex, high-dimensional representations directly from image data. Conditional generative adversarial networks (cGANs)^28,29^ can learn mappings between imaging domains without the need for explicit physical modelling, making them well suited to complex or unknown transformation processes. Recent work has demonstrated the feasibility of transforming plasma emission images into representations of the underlying surface structure.^30,31^

Here, we show that a single-shot laser-induced plasma emission image can support both pollen morphology reconstruction and apparent hydration state classification. A cGAN trained on paired plasma and darkfield microscope images reconstructs surface structure from perpendicular emission patterns, revealing morphological information beyond the compositional signatures typically accessed in LIBS. Whereas conventional LIBS generally analyses one-dimensional wavelength-resolved emission to infer chemical composition, this study examines the two-dimensional spatial and color distributions of the plasma plume for information associated with pollen morphology and visually assigned apparent hydration state. A direct performance comparison with wavelength-resolved LIBS was not undertaken here, but chromaticity analysis shows statistically significant color shifts associated with visually assigned hydration state. These results position plasma emission sensing as a single-measurement route to pollen morphology inference and physiological classification for high-throughput phenotyping and precision agricultural sensing.

## Methods

### Sample Preparation

Pollen grains from *Ranunculus* flowers collected on University of Southampton campus grounds were deposited onto glass microscope slides (Superfrost Plus, Epredia) by brushing the anther with a cotton applicator.

### Experimental Setup

A Light Conversion Pharos SP laser was used to produce 190 fs, 1 mJ laser pulses with a central wavelength of 1030 nm that were focused using a Nikon 20× objective (TU Plan ELWD, 0.40 NA) to a spot size of ∼30 μm. The pollen covered glass slide was attached to an XYZ motorized translation stage (Zaber, LSM050A-T4) that could be translated by 5 cm in all directions. A Basler acA4112-20uc camera (1914 × 1200, RGB) was used to image the pollen grains along the laser axis, as shown in Figure 1. The sample was illuminated using a fibre-bundle-coupled white-light LED ring mounted around the surface imaging objective, enabling darkfield imaging of the pollen grains. A shutter positioned between the LED source and the fibre-bundle input was used to switch the illumination off during plasma acquisition, improving the clarity of the plasma images. An additional Basler daA1920- 160uc camera (4096 × 3000, RGB) with an Olympus 50× long working distance objective (SLMPLN, 0.35 NA) was used to image the plasma emission from the laser-pollen interaction perpendicular to the laser axis. Experimental automation and data collection were performed in Python 3.8.19 using Visual Studio Code 1.68.0 on a Windows 10 workstation.

**Figure 1:**
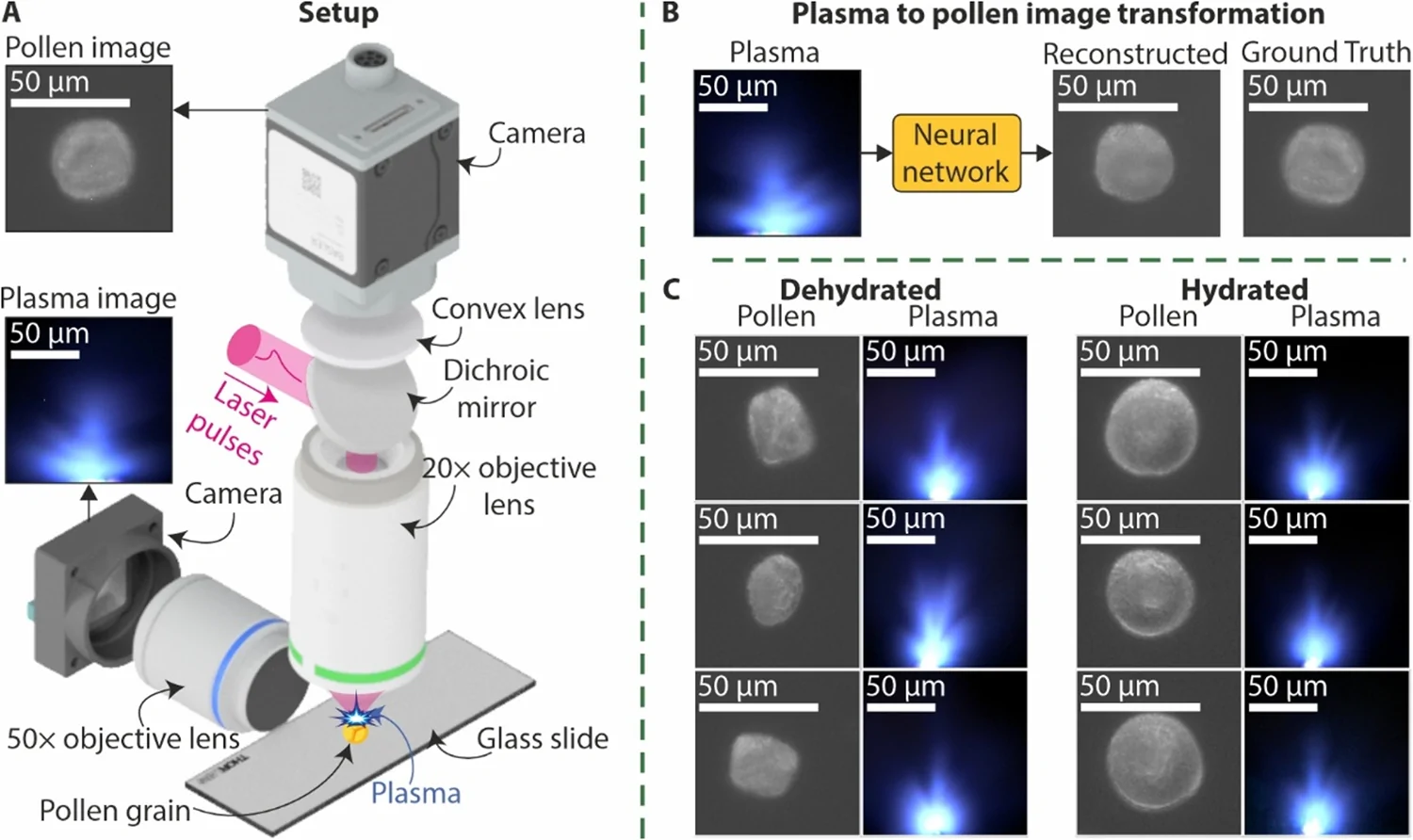
Experimental setup and neural network concept for plasma emission–based pollen imaging. (a) Optical setup for laser-induced plasma generation and imaging of individual pollen grains. (b) Neural network workflow for reconstructing pollen morphology from side-view plasma emission images. The image examples include scale bars based on the corresponding imaging-system calibration. (c) Examples of dehydrated and hydrated pollen used for visual categorization with corresponding plasma emission images.

### Data Collection

Each pollen grain was manually located by moving the motorized translation stages and viewing the surface of the slide with the camera. Upon location, a single pulse was fired at the centre of the pollen grain. An image of the pollen grain was recorded before ablation, and the same location was imaged after to confirm ablation of the pollen grain and its removal from the position. An image of the plasma was recorded upon firing the laser pulse at the pollen grain. The firing of the laser pulse, shutter control and collection of the images was carried out automatically upon keyboard request. Each plasma measurement was generated using a single 190 fs laser pulse. Both cameras used an integration time of 80 ms. The plasma-emission data for an individual grain were therefore obtained from one laser event within a single camera-acquisition interval. Since the laser interaction was destructive, each pollen grain was interrogated only once by the laser, after which the sample was translated to a new position. The total time from laser firing to image saving was approximately 0.5 s, while acquisition of the complete before-ablation, plasma-emission, and after-ablation image sequence required approximately 6.5 s per pollen grain.

A total of 1,020 pollen grains were imaged and ablated, of which 84 were visually assigned to two apparent hydration-state groups, with 42 grains in each group. Larger, approximately spherical grains were assigned to the hydrated-appearing group, whereas smaller, irregularly shaped grains were assigned to the dehydrated-appearing group. These assignments were based on preliminary microscopy and comparison with reference images of hydrated and dehydrated pollen morphology available through the PalDat database.^32^ The labels were not based on independent measurements of water content and should therefore be interpreted as visually assigned apparent hydration states.

### Neural Network

An image-to-image neural network, based on the pix2pix architecture,^28,33^ was used to convert side-view images of the plasma into top-down surface images of the pollen grains. Each plasma image was paired with its corresponding pollen image, and both were cropped and resized to 256 × 256 × 3 pixels. The network comprised a depth-six U-Net generator and a depth-four PatchGAN discriminator. The generator was trained using adversarial cross-entropy loss combined with L1 reconstruction loss weighted by a factor of 100. The generator and discriminator were optimized using Adam^34^ with learning rates of 2 × 10^–4^, β_1_ values of 0.5, and β_2_ values of 0.999. The discriminator loss was weighted by a factor of 0.5. The network was trained for 270 epochs, at which point the generator loss had plateaued, using a minibatch size of eight. A total of 1,000 paired images were used for training, and 20 paired images were retained for testing. The neural network was trained in MATLAB R2024a on a Dell Precision 7865 workstation running Windows 11 Enterprise, equipped with two NVIDIA RTX A6000 GPUs, each with 48 GB of GDDR6 memory, 128 GB of system memory, and a 32-core AMD Ryzen Threadripper PRO 5975WX processor operating at 3.60 GHz. Once trained the neural network took approximately 100 ms to process a plasma image and output a reconstructed image of the pollen grain.

### Chromaticity Analysis and Classification

Plasma emission color was quantified using chromaticity coordinates defined as *r* = *R*/(*R* + *G* + *B*) and *g* = *G*/(*R* + *G* + *B*). Histogram-based chromaticity features and scalar color metrics were extracted for each sample. For each segmented plasma image, the pixel-level *r-g* distribution was represented using a normalised 16 × 16 histogram, providing 256 features. Ten scalar color features were additionally calculated: mean red, green, and blue intensity; mean *r*, *g*, and *b* chromaticity; blue-to-red intensity ratio; normalised yellow index; mean HSV saturation; and mean HSV value. The combined feature vector therefore contained 266 features.

A linear SVM was implemented using “fitcsvm” in MATLAB R2024a. A linear SVM was selected because the dataset contained 84 observations and the feature representation was comparatively high-dimensional. This provided a relatively simple, regularised classifier with fewer fitted parameters than more complex ensemble or deep-learning models. Performance was evaluated using leave-one-out cross-validation because the dataset comprised 84 individual grains. In each fold, one grain was reserved for testing and the remaining 83 grains were used for model fitting, such that every pollen grain served once as an unseen test sample. This approach maximised the training data available in each fold while preventing the test grain from being used to fit the classifier. Any feature standardization and model fitting were performed independently within each fold to avoid information leakage. Performance was reported using accuracy, sensitivity, specificity, precision, F1 score, balanced accuracy, Matthews correlation coefficient, and an exact 95% binomial confidence interval. Statistical significance was assessed using 1,000 random permutations of the group labels, with the complete leave-one-out procedure repeated for each permutation. The corrected permutation probability was calculated as [1 + *∑(A*_perm_ *≥ A*_obs_)]/*(N*_perm_ +1), where *A*_obs_ is the observed classification accuracy, *A*_perm_ is the classification accuracy obtained for a permuted dataset, and *N*_perm_ is the number of permutations.

## Results and Discussion

### Pollen Morphology Reconstruction

Single-shot plasma emission images were used as inputs to the image translation neural network to reconstruct the morphology of individual pollen grains, with representative results shown in Figure 2, where side-view plasma emissions are mapped to reconstructed top-down microscope images of the pollen. Each column represents a different pollen grain, with the corresponding plasma image (row 1), experimental ground truth pollen image (row 2), reconstructed pollen image (row 3), and the difference between ground truth and reconstructed images (row 4), where yellow indicates greater differences in pixel intensity. The reconstructed outputs closely resemble the ground truth pollen images, capturing overall size and shape. Differences between reconstructed and ground truth images are primarily localized to edges and fine details, as highlighted in the difference maps. Errors were concentrated around pollen grain boundaries because small spatial differences between sharp edges produce large local pixel differences. In addition, the Gaussian beam profile concentrates the highest fluence and strongest ablation response near the beam centre, potentially providing less information about peripheral features. The indirect mapping from the broadened side-view plasma image to the top-view pollen image likely further limits the recovery of fine boundary structure, since the ground-truth image is a direct top-down darkfield measurement containing sharp boundaries and surface-intensity variations, whereas the network input is a perpendicular image of the spatially expanding plasma, with information integrated along the camera line of sight. Fine features in the darkfield image may therefore not produce unique or measurable changes in the plasma plume and are thus difficult to reconstruct. Consequently, the network recovers the overall particle extent and gross morphology more reliably than edge texture and small surface features.

**Figure 2:**
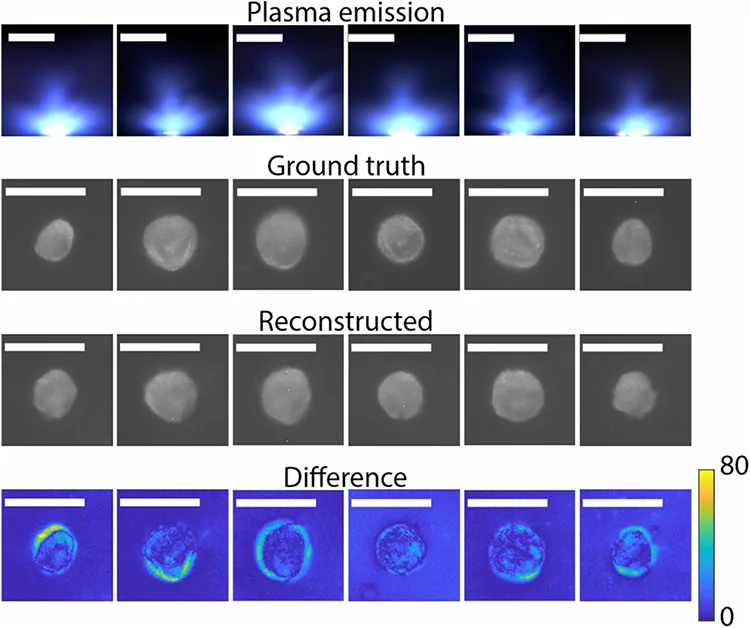
Reconstruction of pollen morphology from plasma emission. Representative examples showing plasma emission images (1^st^ row), corresponding ground truth optical images of pollen grains (2^nd^ row), reconstructed images generated by the neural network (3^rd^ row) and absolute difference maps (4^th^ row) highlight reconstruction errors, which are primarily localized to edges and fine features. Scale bars in all images correspond to 50 μm.

For quantitative reconstruction evaluation, each ground-truth pollen image was smoothed and thresholded to identify the largest connected pollen region. A pollen-centered region of interest was defined using the resulting bounding box with a 10-pixel margin and the same region was applied to the ground-truth image, cGAN reconstruction, and mean-image baseline. Structural similarity index (SSIM), peak signal-to-noise ratio (PSNR), and mean absolute error (MAE) were then calculated from greyscale-converted image pixels within this region rather than from the thresholded images. As displayed in Table 1, across the complete 20-image test set, the cGAN achieved an SSIM of 0.794 ± 0.054, a PSNR of 23.91 ± 2.22 dB, and an MAE of 11.70 ± 3.31. An input-independent baseline, constructed by averaging the 1,000 training targets and using the resulting mean image as the prediction for every test sample, achieved an SSIM of 0.831 ± 0.056, a PSNR of 23.67 ± 2.62 dB, and an MAE of 12.65 ± 4.23. The cGAN produced a higher PSNR and lower MAE than the baseline, whereas the smooth mean-image baseline produced a higher SSIM. The SSIM difference was statistically significant (*p* = 8.86 × 10^–5^), whilst the differences in PSNR (*p* = 0.576) and MAE (*p* = 0.332) were not statistically significant. The higher baseline SSIM is attributed to the smooth average structure and common spatial alignment of the training targets. In contrast, the cGAN outputs varied between plasma inputs and reproduced sample-dependent differences in pollen dimensions and outline.

**Table 1:** Quantitative Reconstruction Performance of the cGAN and Mean-Image Baseline across the Test Set

Method	SSIM	PSNR (dB)	MAE
Mean training-image baseline	0.831 ± 0.056	23.67 ± 2.62	12.65 ± 4.23
cGAN reconstruction	0.794 ± 0.054	23.91 ± 2.22	11.70 ± 3.31
*p*-value	8.86 × 10^–5^	0.576	0.332

To determine whether the cGAN captured sample-specific morphological variation, equivalent diameter and projected area were extracted from the reconstructed and ground-truth images. Reconstructed equivalent diameter was strongly correlated with ground-truth equivalent diameter, with *r* = 0.859 and *p* = 1.28 × 10^–6^ (see Figure 3), while the reconstructed projected area was similarly correlated with ground-truth projected area, with *r* = 0.851 and *p* = 2.00 × 10^–6^. These correlations demonstrate that the cGAN captured sample-dependent variation in gross pollen dimensions, whereas the fixed mean-image baseline necessarily predicted the same dimensions for every input. Outline agreement was also high, with a Dice coefficient of 0.905 ± 0.046 and an intersection over union of 0.830 ± 0.071 for the cGAN, compared with 0.886 ± 0.066 and 0.800 ± 0.098 for the mean-image baseline. The differences were not statistically significant for Dice or intersection over union.

**Figure 3:**
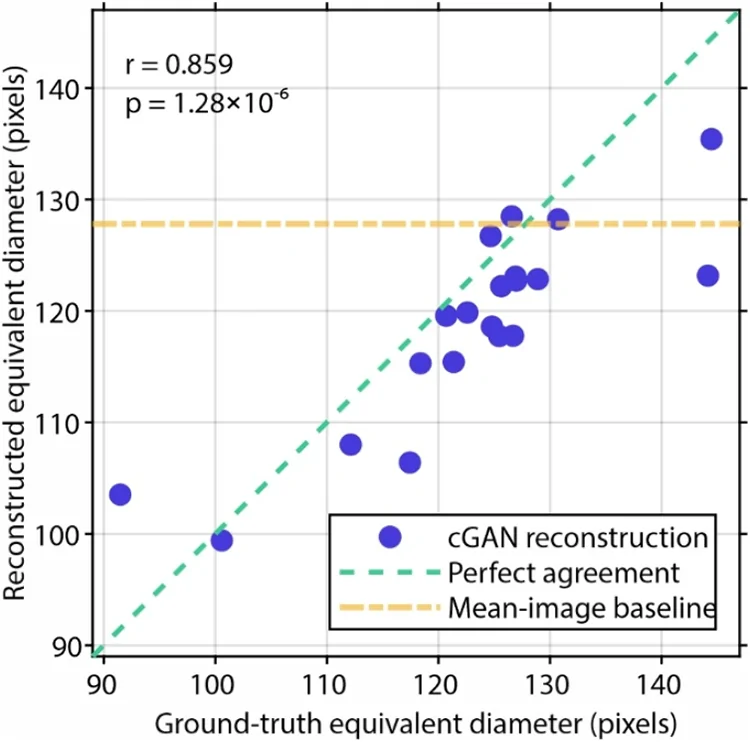
Sample-specific morphological agreement between cGAN reconstructions and ground-truth pollen images. Correlations between reconstructed and ground-truth equivalent diameter and projected area across the 20-image test set. Green dashed lines indicate perfect agreement, and yellow horizontal lines indicate the fixed predictions of the mean-image baseline.

### Hydration State Determination

Building on the ability to reconstruct pollen morphology from plasma images, we next examined whether plasma emission also contains information beyond structural features, namely the visually assigned hydration state. It was found that plasma emission color differs systematically between these groups, forming distinct clusters in chromaticity space (Figure 4). Hydrated-appearing grains exhibited lower mean *r* and *g* chromaticity than dehydrated-appearing grains (*r* = 0.192 ± 0.015 vs 0.203 ± 0.016, *p* = 0.003; *g* = 0.251 ± 0.023 vs 0.265 ± 0.022, *p* = 0.006), demonstrating an association between plasma colour and the visually assigned groups. The chromaticity difference may arise however from hydration-associated changes in pollen morphology, laser coupling, ablated mass, plasma conditions, and plume expansion. Since the RGB measurements do not resolve individual emission wavelengths and no independent measurements of pollen water content were carried out, the observed shift cannot be attributed uniquely to water content.

**Figure 4:**
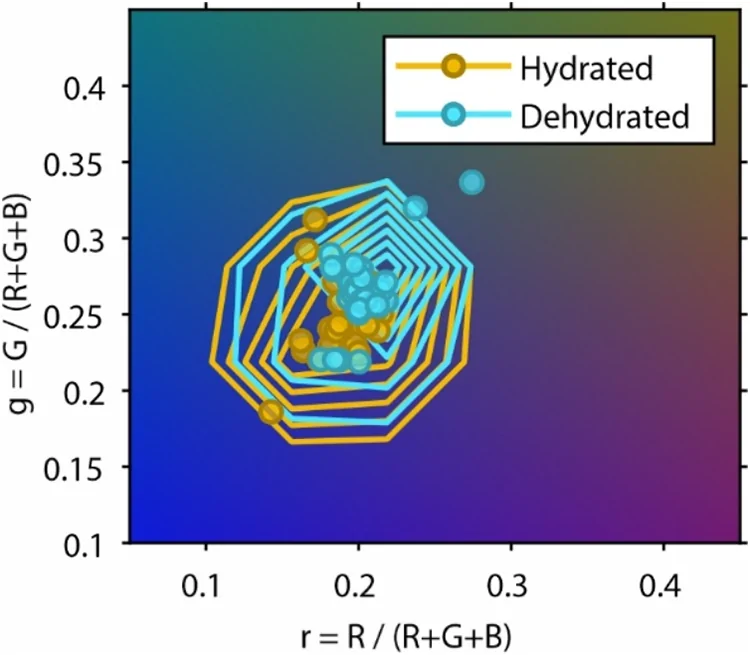
Plasma emission chromaticity for visually assigned hydrated and dehydrated pollen grains. Individual points show chromaticity centroids from single pollen measurements, and contours show mean chromaticity histograms for each group in RGB chromaticity space.

To assess the practical separability of these features, a simple SVM analysis based on chromaticity histogram and scalar colour features was applied to the dataset and correctly identified 64 out of 84 samples, corresponding to an accuracy of 76.2% and an exact 95% confidence interval of 65.7–84.8%, with statistically significant separation (*p* = 0.001). Treating the visually assigned apparent hydrated group as the positive class, the classifier produced 29 true positives, 35 true negatives, 7 false positives, and 13 false negatives. Sensitivity was 69.0%, specificity was 83.3%, precision was 80.6%, the F1 score was 74.4%, balanced accuracy was 76.2%, and the Matthews correlation coefficient was 0.529.

Because the dataset was balanced, with 42 grains in each group, the expected majority-class or random-class baseline accuracy was 50%. The SVM therefore exceeded this baseline by 26.2 percentage points. Although the performance was significantly above chance, the classification errors and 95% confidence interval indicate appreciable uncertainty and overlap between the groups. The result is therefore interpreted as proof of concept for detecting plasma-colour differences associated with visually assigned apparent hydration state, rather than as quantitative measurement of pollen water content.

The chromaticity shift represents a change in the broad RGB-camera response and cannot be attributed to a specific emission feature. The classification therefore demonstrates discrimination between visually assigned groups rather than validated quantitative hydration sensing. Future work combining controlled-humidity measurements with wavelength- and time-resolved spectroscopy should investigate the physical origin of the observed color differences.

All measurements were acquired under fixed nominal laser, imaging, and laboratory conditions. The present dataset was not designed to quantify sensitivity to variation in pulse energy, focal position, temperature, relative humidity, pressure, or ambient gas composition. These factors could influence the amount of ablated material, plasma temperature and electron density, plume dimensions, emission intensity, and recorded chromaticity. However, normalization of each chromaticity histogram by its total count reduces direct dependence on the number of segmented plasma pixels and overall plume area. It does not, however, guarantee invariance to changes in laser–pollen coupling or plasma-emission behaviour. The reported performance therefore applies to the experimental conditions investigated here. Systematic perturbation studies and external validation will be required before deployment under variable laboratory or field conditions.

## Conclusion

We have demonstrated that single-shot laser-induced plasma emission images can support pollen morphology reconstruction and apparent hydration state classification. Using deep learning and chromaticity analysis, plasma emission was shown to contain structural and physiological information beyond conventional compositional readouts. More specifically, by combining deep learning with two-dimensional plasma-emission imaging, we generated top-view representations of pollen grains from their side-view plasma-emission patterns. This approach offers a route toward rapid, automated pollen phenotyping, with future work extending the method to additional species and field-deployable systems.

## Data Availability

Data underlying the results presented in this paper are available in ref^35^
